# Machine Learning Model Based on Radiomic Features for Differentiation between COVID-19 and Pneumonia on Chest X-ray

**DOI:** 10.3390/s22176709

**Published:** 2022-09-05

**Authors:** Young Jae Kim

**Affiliations:** Department of Biomedical Engineering, Gachon University, 21, Namdong-daero 774 beon-gil, Namdong-gu, Inchon 21936, Korea; youngjae@gachon.ac.kr; Tel.: +82-32-458-2844; Fax: +82-32-460-2361

**Keywords:** COVID-19, pneumonia, radiomic feature, machine learning, chest X-ray

## Abstract

Machine learning approaches are employed to analyze differences in real-time reverse transcription polymerase chain reaction scans to differentiate between COVID-19 and pneumonia. However, these methods suffer from large training data requirements, unreliable images, and uncertain clinical diagnosis. Thus, in this paper, we used a machine learning model to differentiate between COVID-19 and pneumonia via radiomic features using a bias-minimized dataset of chest X-ray scans. We used logistic regression (LR), naive Bayes (NB), support vector machine (SVM), k-nearest neighbor (KNN), bagging, random forest (RF), extreme gradient boosting (XGB), and light gradient boosting machine (LGBM) to differentiate between COVID-19 and pneumonia based on training data. Further, we used a grid search to determine optimal hyperparameters for each machine learning model and 5-fold cross-validation to prevent overfitting. The identification performances of COVID-19 and pneumonia were compared with separately constructed test data for four machine learning models trained using the maximum probability, contrast, and difference variance of the gray level co-occurrence matrix (GLCM), and the skewness as input variables. The LGBM and bagging model showed the highest and lowest performances; the GLCM difference variance showed a high overall effect in all models. Thus, we confirmed that the radiomic features in chest X-rays can be used as indicators to differentiate between COVID-19 and pneumonia using machine learning.

## 1. Introduction

Currently, for the diagnosis of COVID-19, real-time reverse transcription polymerase chain reaction (RT-PCR) is used as the standard diagnostic test [1]. However, it is time-consuming and requires expensive dedicated equipment [2]. Hence, imaging procedures can be a viable alternative to RT-PCR [3,4]. In particular, chest X-rays can significantly speed up screening for COVID-19 as they are inexpensive and can rapidly evaluate the condition of the lungs. However, diagnosis is dependent on the skill of the doctor, and differentiating it from pneumonia, which has similar symptoms, is difficult [5,6].

Machine learning can help differentiate between COVID-19 and pneumonia by analyzing differences in the scans that are difficult to detect with the naked eye [7]. Recently, many studies have reported the application of machine learning for COVID-19 diagnosis. Its objective analysis can help the medical staff screen for COVID-19 rapidly. In 2021, Hasoon et al. implemented machine learning using six models (LBP-KNN, HOG-KNN, Haralick-KNN, LBP-SVM, HOG-SVM, and Haralick-SVM) on 5000 X-ray images of normal and COVID-19-infected lungs, and published it on GitHub. The LBP-KNN model reported an average accuracy of 98.66% when performing classification of the normal and infected scans [8]. In 2021, Kassania et al. collected X-ray images of 137 COVID-19 patients and 137 normal individuals published on Github and Kaggle, respectively, and extracted features using eight convolutional neural network (CNN)-based architectures (MobileNet, DenseNet, Xception, ResNet, InceptionV3, InceptionResNetV2, VGGNet, and NASNet). Then, the normal and COVID-19 scans were classified using six machine learning models (LightGBM, bagging tree, Adaboost, random forest, XGBoost, and decision tree). Consequently, the bagging tree model using the features extracted by DenseNet121 showed the best performance, with 99% classification accuracy [9]. In 2021, Jain et al. collected 6432 chest X-ray images (normal, n = 1583; COVID-19, n = 576; pneumonia, n = 4273), published on Kaggle, to classify normal, COVID-19, and pneumonia scans using three deep learning architectures (Inception V3, Xception, and ResNet). Consequently, the Xception model showed the best performance, with 97.97% classification accuracy [10]. In 2021, Sitaula et al. proposed a model combining the attention module and VGG-16 to classify normal, COVID-19, and pneumonia on X-rays for three public datasets. Consequently, the proposed model showed 79.58%, 85.43%, and 87.49% accuracy in three types of public open data, respectively, and the best performance compared to other models reported on the same data [11].

Most of the previous studies aimed to differentiate between normal and COVID-19 scans. Additionally, most of the previous studies that differentiated between COVID-19 and pneumonia scans applied deep learning. Since the lungs of normal patients are clean, it is not difficult to distinguish them from COVID-19 with the naked eye. However, it is difficult to distinguish the lungs of pneumonia patients from COVID-19 visually. Therefore, there is a need for a method to help distinguish between COVID-19 and pneumonia. Deep learning requires a large amount of data for training. However, since the collection of scans is limited, explaining the results of the model is difficult due to the black box feature [12]. In addition, most of the preceding studies used public datasets that often used data from various sources. If a public dataset is used, reliability of the images decreases. The image quality and shooting conditions are often different and COVID-19 diagnosis verification via RT-PCR or clinical diagnosis is uncertain. In particular, the pneumonia scans in most studies used images from collected sources different than the COVID-19 scans. Since the difference in patterns between COVID-19 and pneumonia is not large, a risk of generating bias due to datasets from different sources exists.

In this paper, the X-ray scans of patients whose COVID-19 diagnosis has been verified by RT-PCR diagnosis and of patients who tested positive for pneumonia were collected from a single institution. Using a reliable, bias-minimized dataset, we aimed to find radiomic features that can help differentiate between COVID-19 and pneumonia. In addition, by training and validating various machine learning models based on radiomic features, we have attempted to confirm the applicability of radiomic features and machine learning for COVID-19 screening.

## 2. Materials and Methods

### 2.1. Ethics Statement

This retrospective study was approved by the Institutional Review Board (IRB) of Gil Medical Center with a waiver of the requirement for patients’ informed consent (approval number: GBIRB2020-370).

### 2.2. Data Collection

In this study, chest X-ray images of 250 patients diagnosed with COVID-19 and of 250 patients diagnosed with pneumonia at the Gachon University Gil Medical Center were collected over the period of January 2020 to December 2021. The collected anteroposterior (AP) X-rays were saved in the DICOM format. The COVID-19 scans were collected from 109 males and 141 females with a mean age of 61.58 ± 18.93 years for males (range: 13–100 years) and 64.32 ± 20.06 years for females (range: 6–97 years). Similarly, the pneumonia scans were collected from 142 males and 108 females with a mean age of 68.40 ± 16.80 years for males (range: 23–98 years) and 74.67 ± 16.51 years for females (range: 23–97 years). Table 1 shows the clinical characteristics of the COVID-19 and pneumonia patients.

**Figure 1:**
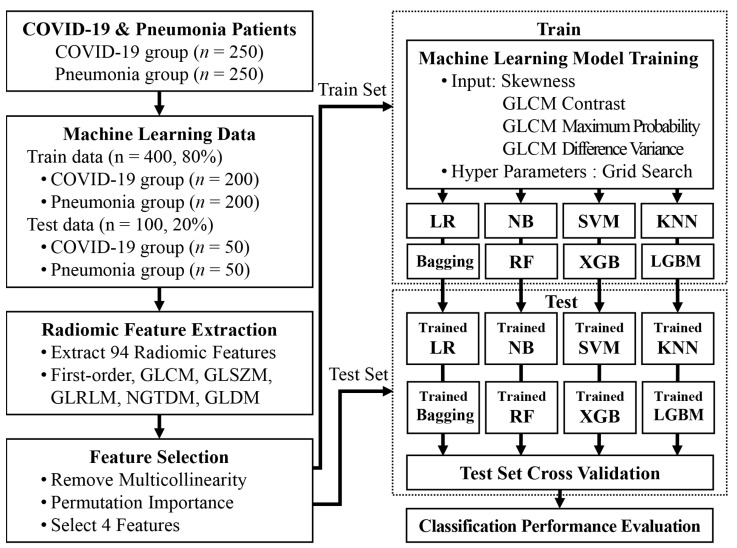
Flowchart showing the data and machine learning process used to differentiate between COVID-19 and pneumonia using chest X-ray images. GLCM, gray level co-occurrence matrix; GLSZM, gray level size zone matrix; GLRLM, gray level run length matrix; NGTDM, neighboring gray-tone difference matrix; GLDM, gray level dependence matrix; LR, logistic regression; NB, naive Bayes; SVM, support vector machine; KNN, k-nearest neighbor; RF, random forest; XGB, extreme gradient boosting; LGBM, light gradient boosting machine.

The collected data were divided into the training and test data for the training and validation of the machine learning model, respectively. For both COVID-19 and pneumonia, 80% of the data were randomly selected as the training data (COVID-19, n = 200; pneumonia, n = 200), and the remaining 20% were used as the test data (COVID-19, n = 50; pneumonia, n = 50). Figure 1 shows the flow chart of the collected data and the machine learning process.

### 2.3. Region of Interest

**Figure 2:**
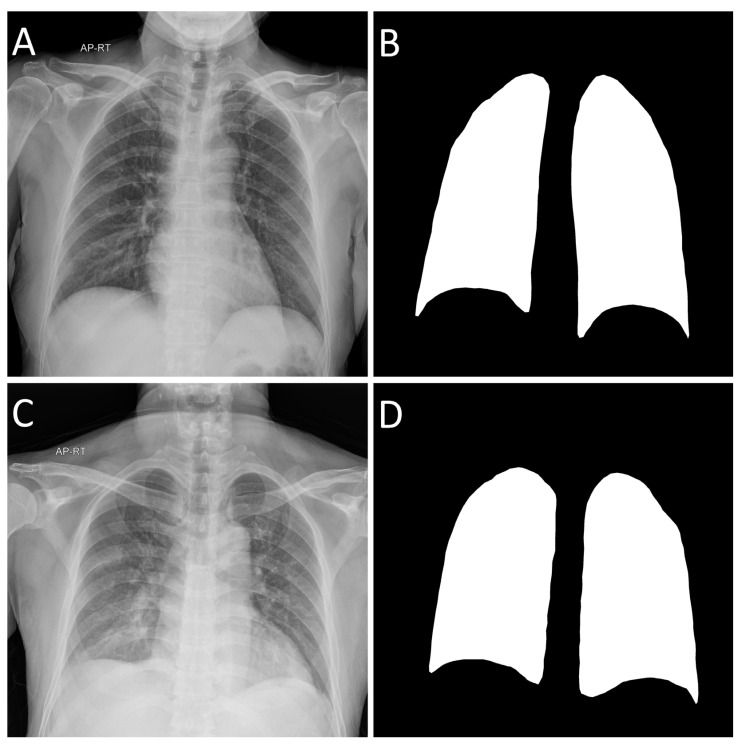
Chest X-rays and ROIs for lungs collected for COVID-19 and pneumonia. (**A**) COVID-19 image, (**B**) ROI mask image from COVID-19 image, (**C**) pneumonia image, and (**D**) ROI mask image from pneumonia image. ROI, region of interest.

To limit the analysis to the lungs, we defined a region of interest (ROI) for each X-ray image. ImageJ (version 1.53e, National Institutes of Health, Bethesda, MD, USA), an open-source software developed by the National Institute of Health (NIH), was used to define the ROI. The boundaries of the left and right lung regions were drawn, excluding the aorta and heart. The drawn ROI was collected by filling the inside and converting it into a binary mask image. Figure 2 shows an example of the ROI for the collected lung region.

### 2.4. Extraction of Radiomic Features

Radiomic features were extracted from the ROI of each image using the Pyradiomics library (version 3.6.2, https://github.com/Radiomics/pyradiomics.git, accessed on 11 July 2022) [13]. To extract the radiomic features, first-order and texture analyses to explain the distribution of the pixel values and pattern of the image surface were used, respectively [14]. First-order analysis extracts statistical features from a histogram of pixel values, and texture analysis extracts features by composing the relationship between the pixels into a matrix [15,16,17]. Five matrix construction methods (gray level co-occurrence matrix (GLCM), gray level size zone matrix (GLSZM), gray level run length matrix (GLRLM), neighboring gray-tone difference matrix (NGTDM), and gray level dependence matrix (GLDM)) were used. GLCM quantifies the spatial relationship of the pixels as a matrix by combining two adjacent pixels [18]. GLSZM quantifies the size of adjacent pixels with the same gray level intensity value as a matrix [19]. GLRLM quantifies the length of consecutive pixels with the same gray level intensity as a matrix for a defined direction [20]. NGTDM quantifies the difference between a specific gray level intensity value and an average intensity value of adjacent pixels in a matrix [21]. GLDM quantifies the relative frequency of gray level intensity values between pixels separated by a defined distance in a matrix [22].

In this study, 19, 24, 16, 16, 5, and 14 features were extracted using first-order analysis, GLCM, GLSZM, GLRLM, NGTDM, and GLDM, respectively. Accordingly, a total of 94 radiomic features were used.

### 2.5. Feature Selection

All the features used in this paper were calculated for different purposes with different formulae. However, if some variables have a high degree of correlation with other variables, data analysis may be negatively affected. To minimize this effect, we eliminated features with variance inflation factors (VIF) greater than 10 via multicollinearity analysis [23].

**Figure 3:**
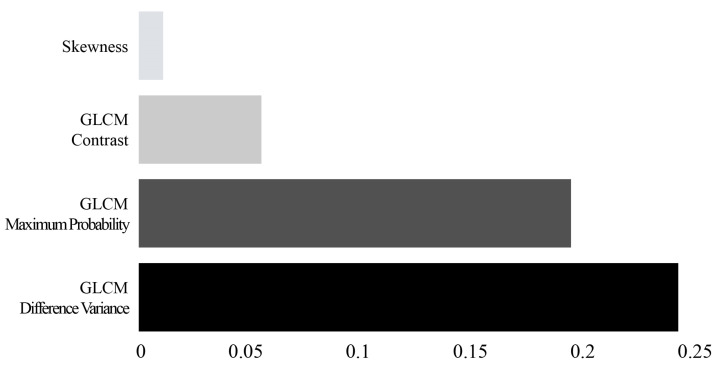
A feature importance plot showing the relative importance of the four final features for the identification of COVID-19 and pneumonia groups. GLCM, gray level co-occurrence matrix.

When input variables larger than the capacity of the capacity are used, the risk of overfitting increases due to the high complexity of the model. Hence, to reduce the number of input variables, after calculating the importance of each feature using the permutation feature importance method, four final input variables were selected among the features with high importance. Permutation feature importance is a method of calculating the importance of components by calculating the change in a specific score when the indices of each feature are randomly shuffled during initial model training [24,25]. The four input variables selected are skewness, GLCM maximum probability, GLCM contrast, and GLCM difference variance, as shown in Figure 3.

### 2.6. Machine Learning Models to differentiate between COVID-19 and Pneumonia

In this study, logistic regression (LR), naive Bayes (NB), support vector machine (SVM), k-nearest neighbor (KNN), bagging, random forest (RF), extreme gradient boosting (XGB), and light gradient boosting machine (LGBM) were used to differentiate between COVID-19 and pneumonia based on the training data. Four previously selected features (skewness, GLCM maximum probability, GLCM contrast, and GLCM difference variance) were used as input variables for each machine learning model.

LR is an architecture that predicts the probability of the data belonging to a certain category as a value between 0 and 1 using regression. A higher value indicates a higher probability of belonging to that category [26]. NB is a supervised learning method that is simple, fast, and provides high accuracy as a statistical classification technique based on the Bayes theorem [27]. SVM is an architecture that learns how to classify data into two data groups by measuring the distance of individual data in two categories, locating the center point between the two data groups, and reporting the optimal hyperplane [28]. KNN is a non-parametric delayed learning architecture that uses Euclidean distance to classify by referring to K other data that are close to each other [29]. Bagging is an ensemble learning model that bootstraps data, makes each independent model with the extracted data, and then aggregates and classifies the learning results of each model [30]. RF is an ensemble learning model architecture that constructs multiple decision trees, passes new data points through each tree simultaneously, and votes on the results classified by each tree to obtain the most voted result [31]. XGB is a tree-based ensemble learning model, based on the gradient boost machine (GBM) that addresses the disadvantages of GBM, such as slow execution time and overfitting regularization [32]. LGBM is a tree-based gradient boosting architecture that uses leaf-wise methods to classify with high speed and high accuracy [33].

We used the grid search method to determine the optimal hyperparameters for each machine learning model. Grid search is a search method that finds the most optimal hyperparameter combination among the combinations of candidate groups of potential parameters while sequentially assigning possible values to the hyperparameters [34]. Table 2 shows the hyperparameters for each machine learning model determined using the grid search.

### 2.7. Validation and Statistical Analysis

In this study, 5-fold cross-validation was performed to prevent the overfitting of each trained machine learning model and secure sufficient test data [35]. Learning and verification were performed five times, and all data were used for verification once. For the verification of discrimination performance, accuracy, sensitivity, specificity, positive predictive value (PPV), and negative predictive value (NPV) were used as indicators. Each performance index was calculated based on true positive (TP), false positive (FP), false negative (FN), and true negative (TN). Accordingly, TP is when COVID-19 is correctly identified, FP is when COVID-19 is misidentified as pneumonia, FN is when pneumonia is misidentified as COVID-19, and TN is when pneumonia is correctly identified. In addition, the area under the receiver operating characteristic (ROC) curve (area under the curve (AUC)) was used to evaluate the predictive performance of each machine learning model [36]. The statistical significance was set as *p* < 0.05.

The software and libraries used for statistical analysis and performance verification were Python (version 3.7.0, Python Software Foundation, Wilmington, DE, USA), sci-kit-learn library (version 0.23.2), MedCalc (version 14.0, MedCalc Software Ltd., Mariakerke, Belgium), and SPSS (version 20, IBM Corp., Armonk, NY, USA).

## 3. Results

**Figure 4:**
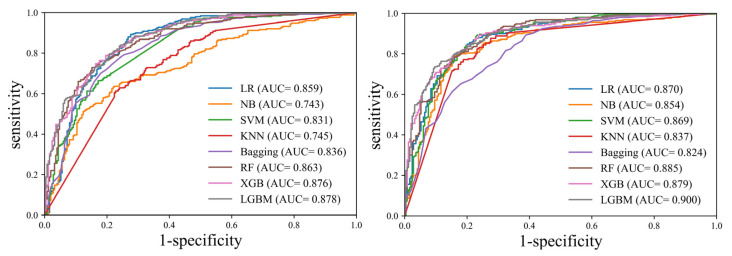
Comparison of ROC curves of the various machine learning models. Left graph: ROC curves without feature selection. Right graph: ROC curves with feature selection. LR, logistic regression; NB, naive Bayes; SVM, support vector machine; KNN, k-nearest neighbor; RF, random forest; XGB, extreme gradient boosting; LGBM, light gradient boosting machine; ROC, receiver operating characteristic; AUC, area under the curve.

In this study, the identification performance of COVID-19 and pneumonia was compared with separately constructed test data for four types of machine learning models trained using skewness, GLCM maximum probability, GLCM contrast, and GLCM difference variance as input variables. Table 3 and Figure 4 show the identification performance of each machine learning model with and without feature selection. When the feature selection step was not performed, all features were used as input data, and when the feature selection step was performed, four selected features were used as input data.

As a result of performance verification for identification between COVID-19 and pneumonia, it showed higher performance in all models except the bagging model when the feature selection step was performed than when the feature selection step was not performed. The bagging model showed AUC = 0.836 (CI: 0.801–0.868) and AUC = 824 (CI: 0.787–0.856) performance with and without feature selection, respectively, and there was no statistically significant difference (*p* = 0.335).

When the feature selection step was performed, the LGBM model showed the highest performance, with AUC = 0.900 (CI: 0.870–0.925), and the bagging model showed the lowest performance, with AUC = 0.824 (CI: 0.787–0.856). The AUCs of each machine learning model had a significant difference (*p* < 0.001).

The results of accuracy performance verification for differentiation between COVID-19 and pneumonia are as follows: The RF model had accuracy = 0.808 (CI: 0.771–0.842), sensitivity = 0.816 (CI: 0.762–0.862), specificity = 0.800 (CI: 0.745–0.848), showing the highest accuracy and balanced performance. The LGBM model showed the second highest accuracy of 0.796 (CI: 0.758–0.831), but showed unbalanced performance with high sensitivity of 0.916 (CI: 0.875–0.947) and low specificity of 0.677 (CI: 0.614–0.734). This implies that LGBM often misidentified pneumonia as COVID-19.

**Figure 5:**
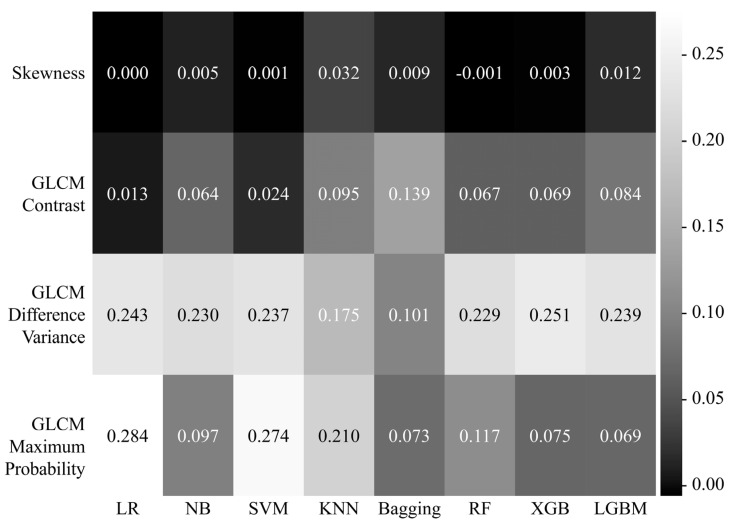
Heatmap showing the effect of the four features on the training of each machine learning model. The higher the heatmap value and the closer to white, the stronger the influence of the feature on the differentiation between COVID-19 and pneumonia. GLCM, gray level co-occurrence matrix; LR, logistic regression; NB, naive Bayes; SVM, support vector machine; KNN, k-nearest neighbor; RF, random forest; XGB, extreme gradient boosting; LGBM, light gradient boosting machine.

Figure 5 shows the heatmap of the influence of the four features used for differentiating between COVID-19 and pneumonia for each machine learning model. In LR, SVM, and KNN, the GLCM maximum probability (LR: 0.284, SVM: 0.274, KNN: 0.210) appeared as the feature having the greatest influence on differentiation performance, but in NB, RF, XGB, and LGBM, the GLCM difference variance (NB: 0.230, RF: 0.229, XGB: 0.251, LGBM: 0.239) was found to be the most influential feature. In particular, the GLCM difference variance (LR: 0.243, NB: 0.230, SVM: 0.237, KNN: 0.175, bagging: 0.101, RF: 0.229, XGB: 0.251, LGBM: 0.239) showed a high overall effect in all models.

## 4. Discussion

In this study, 4 features significant for identification between COVID-19 and pneumonia were selected out of a total of 94 radiomic features extracted from chest X-ray images. Next, using the selected features as input variables, a machine learning model to differentiate between COVID-19 and pneumonia was trained and its performance was verified. Consequently, all machine learning models showed high identification performance with an AUC > 0.8. In particular, the LGBM model showed the highest performance with AUC = 0.900. These results imply that the four significant features, namely skewness, GLCM maximum probability, GLCM contrast, and GLCM difference variance, used as input variables, can be used to differentiate between COVID-19 and pneumonia.

Unlike deep learning, machine learning uses features extracted from the images. Hence, interpreting the results is easy. Essentially, interpreting the effect of each feature in the learned model on the differentiating performance is easy. A small and large value of skewness, a first-order type feature, results in a histogram that is skewed to the right and left, respectively. When the histogram is skewed to the right, the image is generally brighter, and when the histogram is skewed to the left, the image is darker. GLCM contrast, GLCM maximum probability, and GLCM difference variance are the texture features of all the image surfaces and are calculated from the GLCM matrix analyzed from the image. GLCM contrast is a feature for viewing the difference between adjacent gray level intensities. The greater the contrast, the greater the difference between adjacent gray level intensities. GLCM maximum probability is defined as a pair of neighboring gray level intensity values that occur maximally in the GLCM matrix. A greater value indicates more identical patterns in the image. GLCM difference variance is used to measure heterogeneity by assigning more weight as the gray level intensity pairs deviate more from the mean in the GLCM matrix. Therefore, a larger value implies a higher number of texture patterns on the image surface.

The size of the four selected features was determined for each of the COVID-19 and pneumonia groups. Skewness was 0.2948 ± 0.2464 and 0.3224 ± 0.3092 (*p* = 0.272), GLCM contrast was 0.04371 ± 0.01242 and 0.03745 ± 0.02234 (*p* = 0.0001), GLCM maximum probability was 0.005176 ± 0.001932 and 0.007158 ± 0.003697 (*p* = 0.0001), and GLCM difference variance was 1.5758 ± 0.4185 and 1.9410 ± 1.3985 (*p* = 0.0001). GLCM contrast tended to be larger in the COVID-19 group than in the pneumonia group, but the GLCM maximum probability and GLCM difference variance tended to be smaller. With regards to skewness, although the COVID-19 group showed a smaller trend compared to the pneumonia group, it showed no statistically significant difference. Combining these characteristics, we interpreted them as follows: COVID-19 has a rougher surface texture than pneumonia, and pneumonia has a slightly more uniform surface texture compared to COVID-19 but shows various texture patterns. Since pneumonia shows significant consolidation throughout the lungs, various texture patterns appear, and many textures of the same pattern occur simultaneously. For COVID-19, the central part of the lung is black and transparent, but the pattern of clear contrast between the central part of the lung and the lesion increased due to the GGO and consolidation towards the outer part.

In this study, we compared the identification performance with and without feature selection. As a result, feature selection improved the performance in all models except the bagging model. When all features were used as input data without feature selection, too many features seemed to cause overfitting to the training data. Although the bagging model showed the opposite result, there was no significant difference between AUCs, so it was somewhat difficult to interpret whether feature selection affected the performance.

The LGBM model showed the highest AUC but displayed unbalanced performance between sensitivity and specificity. However, sensitivity is very important in clinical practice because the lesion should not be missed. The LGBM model’s performance is disproportionate, but its high sensitivity can be helpful in COVID-19 screening.

In this study, we confirmed that the radiomic features in chest X-rays can be used as indicators to differentiate between COVID-19 and pneumonia using machine learning. However, this study poses a limit for sufficient verification of diagnosis. Since the data used are directly collected from a single institution, the reliability of the data is sufficient. However, for sufficient verification, more data are required, and additional verification of multi-national and multi-institutional data is required. In addition, verifying details according to the severity is necessary because differences in the symptoms and aspects shown in the image depending on the severity of COVID-19 and pneumonia may exist. From an engineering point of view, it is necessary to apply and compare machine learning and deep learning models that are more appropriate for COVID-19 screening through comparison with the various latest models. In the future, if sufficient verification is conducted through various additional experiments, the radiomic features and machine learning in the clinical screening test for COVID-19 using chest X-rays will provide more information to the medical staff when differentiating between COVID-19 and pneumonia.

## 5. Conclusions

In this study, machine learning used radiomic features as a useful indicator to quantify and differentiate between COVID-19 and pneumonia using chest x-rays. If sufficient analysis and validation are conducted through additional studies in the future, the radiomic features could be usefully utilized as non-invasive biomarkers that provide more information in the identification between COVID-19 and pneumonia. In addition, the combination of radiomic features and machine learning is expected to be helpful for the screening and diagnosis of COVID-19.

## Figures and Tables

**Table 1:** Clinical characteristics of COVID-19 and pneumonia patient cohorts.

Characteristic	Number of Patients (%)		*p* Value
	COVID-19 (n = 250)	Pneumonia (n = 250)	
Age (years)	63.12 ± 19.59	71.11 ± 16.93	*p* < 0.001
Sex			
- Males	109 (43.6%)	142 (56.8%)	*p* = 0.004
- Females	141 (56.4%)	108 (43.2%)	*p* = 0.004
Height (cm)	162.30 ± 10.26	161.65 ± 9.96	*p* = 0.497
Weight (kg)	63.59 ± 14.43	58.53 ± 13.49	*p* < 0.001
BMI (kg/m^2^)	23.95 ± 3.81	22.49 ± 4.25	*p* < 0.001
HTN	115 (46.0%)	133 (53.2%)	*p* = 0.128
DM	63 (25.2%)	82 (32.8%)	*p* = 0.076
Smoking status			
- None	177 (70.8%)	182 (72.8%)	*p* = 0.691
- Former smoker	15 (6.0%)	7 (2.8%)	*p* = 0.127
- Current smoker	10 (4.0%)	31 (12.4%)	*p* = 0.001
Symptom			
- Fever	80 (32.1%)	65 (26.0%)	*p* = 0.159
- Cough	85 (34.0%)	34 (13.6%)	*p* < 0.001
- Sputum	45 (18.0%)	43 (17.2%)	*p* = 0.907
- Dyspnea	28 (11.2%)	85 (34.0%)	*p* < 0.001
- Myalgia	33 (13.2%)	0 (0.0%)	*p* < 0.001
- Sore throat	37 (14.8%)	1 (0.4%)	*p* < 0.001

Values are the mean ± standard deviation or counts (proportions). *p*-value: Student *t*-test. BMI, body mass index; HTN, hypertension; DM, diabetes mellitus.

**Table 2:** Hyperparameters used for training each machine learning model.

Model	Hyperparameters
LR	C: 997.265, penalty: L2, random state: 50
NB	var smoothing: 1 × 10^−9^
SVM	C: 998.196, kernel: linear, probability: true, random state: 50
KNN	leaf size: 20, n neighbors: 2, weights: distance
Bagging	max features: 3, max samples: 5, n estimators: 100, random state: 50
RF	max depth: 190, n estimators: 709, random state: 50
XGB	learning rate: 0.068, max depth: 11, n estimators: 100, random state: 50
LGBM	learning rate: 0.05, max depth: 12, min child samples: 10, num leaves: 40, random state: 50

LR, logistic regression; NB, naive Bayes; SVM, support vector machine; KNN, k-nearest neighbor; RF, random forest; XGB, extreme gradient boosting; LGBM, light gradient boosting machine.

**Table 3:** Each machine learning model’s AUC, sensitivity, specificity, PPV, and NPV for differentiating between COVID-19 and pneumonia with and without feature selection.

	AUC (95% CI)	Accuracy (95% CI)	Sensitivity (95% CI)	Specificity (95% CI)	PPV (95% CI)	NPV (95% CI)
(Without Feature Selection)						
LR	0.859 (0.825–0.888)	0.746 (0.706–0.784)	0.980 (0.954–0.994)	0.512 (0.448–0.576)	0.668 (0.639–0.695)	0.962 (0.914–0.984)
NB	0.743 (0.703–0.781)	0.560 (0.515–0.604)	0.960 (0.928–0.981)	0.160 (0.117–0.211)	0.533 (0.518–0.548)	0.800 (0.672–0.887)
SVM	0.831 (0.795–0.863)	0.532 (0.487–0.576)	0.996 (0.978–0.999)	0.068 (0.040–0.107)	0.517 (0.508–0.525)	0.944 (0.695–0.992)
KNN	0.745 (0.704–0.782)	0.682 (0.639–0.723)	0.912 (0.870–0.944)	0.452 (0.389–0.516)	0.625 (0.596–0.652)	0.837 (0.771–0.887)
Bagging	0.836 (0.801–0.868)	0.716 (0.674–0.755)	0.984 (0.960–0.996)	0.448 (0.385–0.512)	0.641 (0.614–0.666)	0.966 (0.913–0.987)
RF	0.863 (0.830–0.892)	0.720 (0.678–0.759)	0.948 (0.913–0.972)	0.492 (0.428–0.556)	0.651 (0.622–0.679)	0.904 (0.846–0.942)
XGB	0.876 (0.843–0.903)	0.792 (0.754–0.827)	0.880 (0.833–0.918)	0.704 (0.643–0.760)	0.748 (0.710–0.784)	0.854 (0.806–0.892)
LGBM	0.878 (0.846–0.906)	0.784 (0.745–0.819)	0.900 (0.856–0.934)	0.668 (0.606–0.726)	0.731 (0.694–0.765)	0.870 (0.820–0.907)
(With Feature Selection)						
LR	0.870 (0.837–0.898)	0.796 (0.758–0.831)	0.860 (0.811–0.901)	0.732 (0.673–0.786)	0.762 (0.722–0.799)	0.839 (0.792–0.877)
NB	0.854 (0.820–0.883)	0.696 (0.654–0.736)	0.952 (0.918–0.975)	0.440 (0.378–0.504)	0.630 (0.603–0.656)	0.902 (0.838–0.942)
SVM	0.869 (0.836–0.897)	0.790 (0.752–0.825)	0.876 (0.829–0.914)	0.704 (0.643–0.760)	0.747 (0.709–0.783)	0.850 (0.802–0.889)
KNN	0.837 (0.802–0.869)	0.682 (0.639–0.723)	0.912 (0.870–0.944)	0.452 (0.389–0.516)	0.625 (0.596–0.652)	0.837 (0.771–0.887)
Bagging	0.824 (0.787–0.856)	0.742 (0.701–0.780)	0.924 (0.884–0.954)	0.560 (0.496–0.623)	0.677 (0.645–0.708)	0.881 (0.825–0.920)
RF	0.885 (0.854–0.912)	0.808 (0.771–0.842)	0.816 (0.762–0.862)	0.800 (0.745–0.848)	0.803 (0.760–0.840)	0.813 (0.769–0.850)
XGB	0.879 (0.848–0.907)	0.790 (0.752–0.825)	0.804 (0.749–0.851)	0.776 (0.719–0.826)	0.782 (0.739–0.820)	0.798 (0.753–0.837)
LGBM	0.900 (0.870–0.925)	0.796 (0.758–0.831)	0.916 (0.875–0.947)	0.676 (0.614–0.734)	0.739 (0.702–0.773)	0.890 (0.841–0.924)

LR, logistic regression; NB, naive Bayes; SVM, support vector machine; KNN, k-nearest neighbor; RF, random forest; XGB, extreme gradient boosting; LGBM, light gradient boosting machine; AUC, area under the curve; PPV, positive predictive value; NPV, negative predictive value; CI, confidence interval.

## Data Availability

The datasets generated or analyzed during the current study are available from the corresponding author upon reasonable request.
